# Allogeneic fibroblasts ameliorate intervertebral disc degeneration by reducing osteophytes in rabbits

**DOI:** 10.3389/fmed.2024.1488727

**Published:** 2024-11-01

**Authors:** Chen Chen, Yizhuo Huang, Lei Shi, Li Zhou, Shenao Zhou, Hongjin Wan, Xiao Yang, Jie Zhao

**Affiliations:** ^1^Shanghai Key Laboratory of Orthopaedic Implants, Department of Orthopaedic Surgery, Shanghai Ninth People’s Hospital, Shanghai Jiao Tong University School of Medicine, Shanghai, China; ^2^Shanghai Jiao Tong University School of Medicine, Shanghai Ninth People’s Hospital, Shanghai, China; ^3^FibroX Therapeutics Inc., Shanghai, China; ^4^Celliver Biotechnology Inc., Shanghai, China

**Keywords:** IVDD, interverbal disc, interverbal disc degeneration, allogeneic, fibroblast

## Abstract

**Introduction:**

Low back pain (LBP) was commonly induced by intervertebral disc degeneration (IVDD), which is accompanied by the loss of disc height and osteophyte generation. Cell-based therapy is a promising treatment for preventing the degeneration of interverbral disc. In our study, allogeneic fibroblasts are shown to ameliorate intervertebral disc degeneration by reducing osteophytes in rabbits.

**Methods:**

We established a rabbits-derived fibroblast (Rab-Fib) which could be expanded in vitro and constructed puncture-induced intervertebral disc degeneration rabbit model. Histologic and imaging examinations and analyses were performed after 2 weeks, 3 months, and 12 months.

**Results:**

Our data indicate that stable and reliably-extracted allogeneic fibroblasts can effectively ameliorate intervertebral disc degeneration by reducing osteophytes.

**Conclusion:**

Our study provides a basis for advancing the further translation of fibroblasts in intervertebral disc therapy.

## 1 Introduction

Low back pain (LBP) induced by the degeneration of intervertebral disc (IVDD) has become a major cause of disability among the senior individuals (1), more than 60% of citizens were once undergone LBP, which brought a heavy burden to the society (2, 3).Currently, as the change of lifestyles, more younger individuals were undergone discogenic pain. However, the treatments for IVDD were still limited as the structure of IVD is special, which is a cartilaginous tissue that connects two adjacent vertebral bodies, in the middle of annulus fibrosus (AF), gel-like nucleus pulposus cells (NPCs) reside in, around which is the cartilaginous endplates (4). In the most of human IVDD cases, the NP tissue architecture deteriorate the first. When NPCs begin to lose its extracellular matrix (ECM) and its ability to maintain hydrostatic pressure. Gradually, with the architectural and structural changes, the height of disc decrease and the stability of spine also reduced (5), leading to spinal instability, back pain and other clinical symptoms (6).

Therefore, preventing the degeneration of IVDs is of a great interest. Cell-based therapy is a promising approach for treating IVDD, as they have the potential to prevent the degeneration of nucleus pulposus cells (NPCs) while maintain the height of IVD. Fibroblast have attracted significant attention in the fields of cell therapy and tissue engineering (7–9). Typically, fibroblasts facilitate tissue repair by promoting cell proliferation and the synthesis of the extracellular matrix (7). Moreover, the growth factors secreted by fibroblasts, as well as those produced by other cells, can stimulate the proliferation of tissue-specific cells, thereby enhancing the body’s natural reparative processes (10, 11). Meanwhile, in clinics, many researches have found that promoting fibrosis in IVD is a necessary for tissue repair and for helping stabilize the spine (12), fibroblast was found that it can integrate into AF and NPCs as well as accelerate fibrosis, leading to heal the disc and stabilize the spine (13).

Therefore, in this study, we explored the therapeutic effects of autologous Rab-Fib transplantation in puncture-induced IVDD rats’ models. The results represented that allogeneic fibroblast stabilized disc height, mitigated disc degeneration, and inhibited osteophyte production.

## 2 Materials and methods

### 2.1 Isolation and cell culture of allogeneic fibroblasts

The rabbit ear-derived skin was collected, washed twice with PBS, immersed in 40 mL of dispase II solution at a concentration of 2 mg/mL, and left at 4°C overnight. The soaked skin tissue was removed to separate the dermis and epidermis, and then the dermis was cut into 1 mm3 pieces, transferred to 15 mL of collagenase XI solution at a concentration of 2 mg/mL, and put into a shaker at 37°C and 200 rpm for digestion for 3.5 h. The resulting 2.2E+07 cells were inoculated into T75 cell culture flasks, 15 mL of complete medium was added, and the cells were placed in a 37°C, 5% carbon dioxide incubator for generation P0. The complete medium was composed of 90% high-sugar DMEM +10% FBS + 1% double antibody +200 units/mL gentamicin.

### 2.2 Expansion culture of rabbit skin fibroblast

When the cell confluence in the cell culture flask reached more than 80%, passaging culture was carried out. After routine trypsin digestion and passaging, all cells in the T75 flasks were digested, centrifuged for cell counting, passaged at a cell ratio of 1:2–1:5, inoculated into T75 cell culture flasks in the order of incremental PX, and placed in an incubator at 37°C with 5% carbon dioxide.

### 2.3 Detection and quality control of fibroblasts

RT–qPCR was performed to detect the gene expression of COL1a1, *α*-SMA, FAP, DDR2, THY-1, TGF-β1, TGF-β2, and TGF-β3 in P5 generation rabbit skin fibroblasts. COL1a1, α-SMA, FAP, DDR2, and THY-1 were used as markers for fibroblast identification; COL1a1, TGF-β1, TGF-β2, and TGF-β3 were used as markers for functional evaluation of fibroblasts.

### 2.4 Cell viability analysis

Cell viability following bleomycin treatment was evaluated using a Cell Counting Kit-8 (CCK-8; Dojindo Laboratories Co., Ltd., Kumamoto, Japan). The cells were seeded onto 96-well plates at a density of 8 × 103 cells/well the day before they were treated with increasing concentrations of bleomycin sulfate (1, 5, or 10 μg/mL, dissolved in PBS; Selleck Chemicals, Houston, TX, United States) for 24, 48, 72, or 96 h. Allogeneic fibroblasts were cultured in DMEM/F12 supplemented with 10% FBS and 1% penicillin/streptomycin. The cell media were changed every 2 days. At the end of the experimental period, the cells were incubated with fresh complete media containing 10 μL of CCK-8 reagent for 1 h at 37°C. Complete media containing CCK-8 reagent but no cells or untreated cells were used as blank and mock controls, respectively. The absorbances [measured as optical density (OD)] at 450 nm were measured on an Infinite M200 Pro multimode microplate reader (Tecan Life Sciences, Männedorf, Switzerland). The ODs of the bleomycin-treated groups were normalized to the corresponding blank ODs to account for background interference.

### 2.5 RNA extraction and real-time quantitative PCR analyses

Total RNA was isolated from tissues and cells using TRIzol Reagent (Thermo Fisher Scientific, Waltham, MA, United States) according to the manufacturer’s protocol. First-strand complementary DNA (cDNA) was reverse transcribed from the extracted RNA using a cDNA synthesis kit (Takara Bio, Otsu, Japan). Relative mRNA expression was determined by RT–PCR using the GoTaq 1-step RT–qPCR System (Promega, Madison, WI, United States) followed by agarose gel electrophoresis (Bio-Rad Laboratories, Hercules, CA, United States). Real-time qPCR was conducted using the TB Green Premix Ex Taq Kit (Takara Bio) on an Applied Biosystems QuantStudio 6 Flex Real-Time PCR System (Thermo Fisher Scientific). Specific primer pairs were designed using NCBI BLAST, and the sequences are provided in Table 1. The gene expression of GAPDH or *β*-actin was used as an internal control. Target gene expression levels were determined using the 2 − ΔΔCT method. The mean CT value of the target genes in the experimental groups was normalized to the CT value of GAPDH or *β*-actin to determine the ΔCT value. This value was then further normalized to that of the control samples to obtain ΔΔCT.

**Table 1:** Primers used in this study for real-time RT–PCR.

Gene	5′-primer-3′
*COL1A1-F*	GCAAGAACGGAGATGACGGAGAAG
*COL1A1-R*	ACCATCCAAACCACTGAAACCTCTG
*α-SMA-F*	GTTGACTGAGGCACCGCTGAAC
*α-SMA-R*	AGTTGTACGTCCAGAGGCATAGAGG
*FAP-F*	TACACAGCAAGTTTCAGCGACTACG
*FAP-R*	CATGAAGGGTGGAAATGGGGAGAC
*THY-1-F*	CCAACTTCACCACCAAGGACGAG
*THY-1-R*	TGTTCTGAGCCAGCAGGTTGATG
*TGF-β1-F*	CATGAACCGACCCTTCCTGC
*TGF-β1-R*	AGTAGTTGGTGTCCAGGGCT
*GAPDH-F*	GTATGATTCCACCCACGGCA
*GAPDH-R*	CCAGCATCACCCCACTTGAT
*S100A4-F*	GGTTGAGTTGGGGAGTGAGT
*S100A4-R*	CGTTGCCTGAGTATTTGTGGA
*FIBRONECTIN-F*	CACAGGGGAAGAAAAGGAGC
*FIBRONECTIN-R*	TGAGTGGATGGGAGGAGAGTC
*VIMENTIN-F*	TGACCGCTTCGCCAACTACA
*VIMENTIN-R*	CGCAACTCCCTCATCTCCTCC
*Desmin-F*	GCCTTGGATGTGGAGATTGC
*Desmin-R*	CCTTTGCTCAGGGCTGGTTT

### 2.6 Animals and surgical procedures

New Zealand white rabbits (Harlan Laboratories, Indianapolis, IN, United States) weighing approximately 2.5 to 3 kg were used in this study (*n* = 24 total). New Zealand rabbits were anesthetized and prepared for skinning in the right lateral position, and a 5-cm incision was made on the iliac spine, touching the transverse process of the lumbar vertebrae to reveal the transverse process along the muscular hiatus and revealing the vertebral body along the anterior edge of the transverse process. The iliac spine was positioned at the inferior intervertebral space, and 2 intervertebral discs were positioned at the superior intervertebral space. The intervertebral disc was punctured with a 20G syringe for 4 mm and then rotated 360° to exit for modeling, and then the cells in the cell treatment group were repunctured (not in the original channel) with a microsyringe to inject 20 μL of cell suspension (1E+06 cells, blowing and mixing before aspirating the cells each time). The degeneration group was injected in the same way with 20 μL of PBS. A630-A631 The inferior intervertebral discs were the cell treatment group, and the superior intervertebral discs were the A632-A635 group. The upper disc discs were the cell treatment group, and the lower disc discs were the degeneration group. An analgesic (buprenorphine HCl 0.01–0.03 mg/kg) was given up to twice daily for 2 to 3 days, when needed, in consultation with the veterinary staff. After recovering from anesthesia, the rabbits returned to their cages and were allowed to acclimate *ad libitum* (8).

### 2.7 Radiographic and magnetic resonance imaging analysis

Digital X-ray imaging of the punctured intervertebral discs was conducted in the anteroposterior axis with a 21-lp/mm detector that provides up to × 5 geometric magnification (Faxitron VersaVision; Faxitron Bioptics LLC, Tucson, AZ, United States). MRI of the same punctured intervertebral discs was carried out on a Siemens Magnetom E11 (Siemens Healthineers, Erlangen, Germany) with the following parameters: TR 3000 ms, TE 80 ms, 1.1 mm thickness, 0.22 mm interval, FOV 160 × 65 mm, and voxel size 0.25 × 0.25 × 1.1 mm. The DHI was expressed as the mean of the 3 measurements from midline to the boundary of the central 50% of disc width divided by the mean of the 2 adjacent vertebral body heights. Changes in the DHI of punctured discs were expressed as a percentage (%DHI = postpunctured DHI/prepunctured DHI × 100) (14). The MRI index values were also measured using Analyze 14.0 software (AnalyzeDirect, Overland Park, KS, United States). These values are the product of the average signal intensity of the NP and area of the NP. We calculated the relative gray value and MRI index of the target IVDs relative to the values of normal IVDs (intact control). The Weishaupt grading system for lumbar facet joint degeneration can be used in CT (15). All image assessments were performed by three independent observers who were blinded to the samples, and the mean of the three evaluations was recorded (Table 2).

**Table 2:** Weishaupt grading system.

Grade 0	Normal facet joint width (2–4 mm)
Grade 1	Facet joint space narrowing, small osteophytes and/or mild articular process hypertrophy
Grade 2	Facet joint space narrowing, moderate osteophytes, moderate articular process hypertrophy and/or small subarticular bone erosions
Grade 3	Facet joint space narrowing, large osteophytes, severe articular process hypertrophy, subarticular bone erosions and/or subchondral cyst formation

### 2.8 Histopathologic analysis

The disc specimens were fixed with formaldehyde, embedded in paraffin, and then cut serially into 5-μm sections. The sections were deparaffinized, rehydrated, and subjected to HE and Safranin O staining and immunohistochemistry. The images were then captured by a microscope and evaluated by histology researchers in a blinded manner. The images were quantified using ImageJ software to determine the percentages of positive cells in the samples. All image assessments were performed by three independent observers who were blinded to the samples, and the mean of the three evaluations was recorded. The tissue was scored according to the histological grading criteria by sive (16). We calculated the percentage of relative the height of disc of the target IVDs relative to the values of normal IVDs (intact control; Table 3).

**Table 3:** Histological grading system of intervertebral disc.

Category	Grade
Annulus fibrosus	1. Annularly arranged fiber ring, without terminations or twists.
	2. Interruption or distortion below 30%.
	3. Interruption or distortion over 30%.
Demarcation of the annulus	1. Clear demarcation fibrosus from nucleus pulposus.
	2. Slight interruption in demarcation.
	3. Heavy integration of demarcation.
Number and morphology of NPCs	1. Normal morphology, rich in NPCs, abundant extracellular matrix.
	2. Slight decrease in the number of NPCs.
	3. Significant cell loss (more than 50%).
Extracellular matrix of NPCs	1. Extracellular matrix in normal gel-like form.
	2. Slight coagulation of extracellular matrix.
	3. Severe coagulation of extracellular matrix.

### 2.9 Statistical analysis

Data analysis was performed using SPSS 22.0 (IBM Corp. Armonk, N.Y.), and the normally distributed data are presented as the mean ± S.E.M. The differences between the two groups were analyzed by using Student’s t test. Comparisons between multiple groups were assessed by using one-way ANOVA followed by a *post-hoc* test (least significant difference). *p* < 0.05 was considered to indicate a statistically significant difference between groups.

## 3 Results

### 3.1 Preparation and growth characteristics of rabbit allogeneic fibroblasts

**Figure 1:**
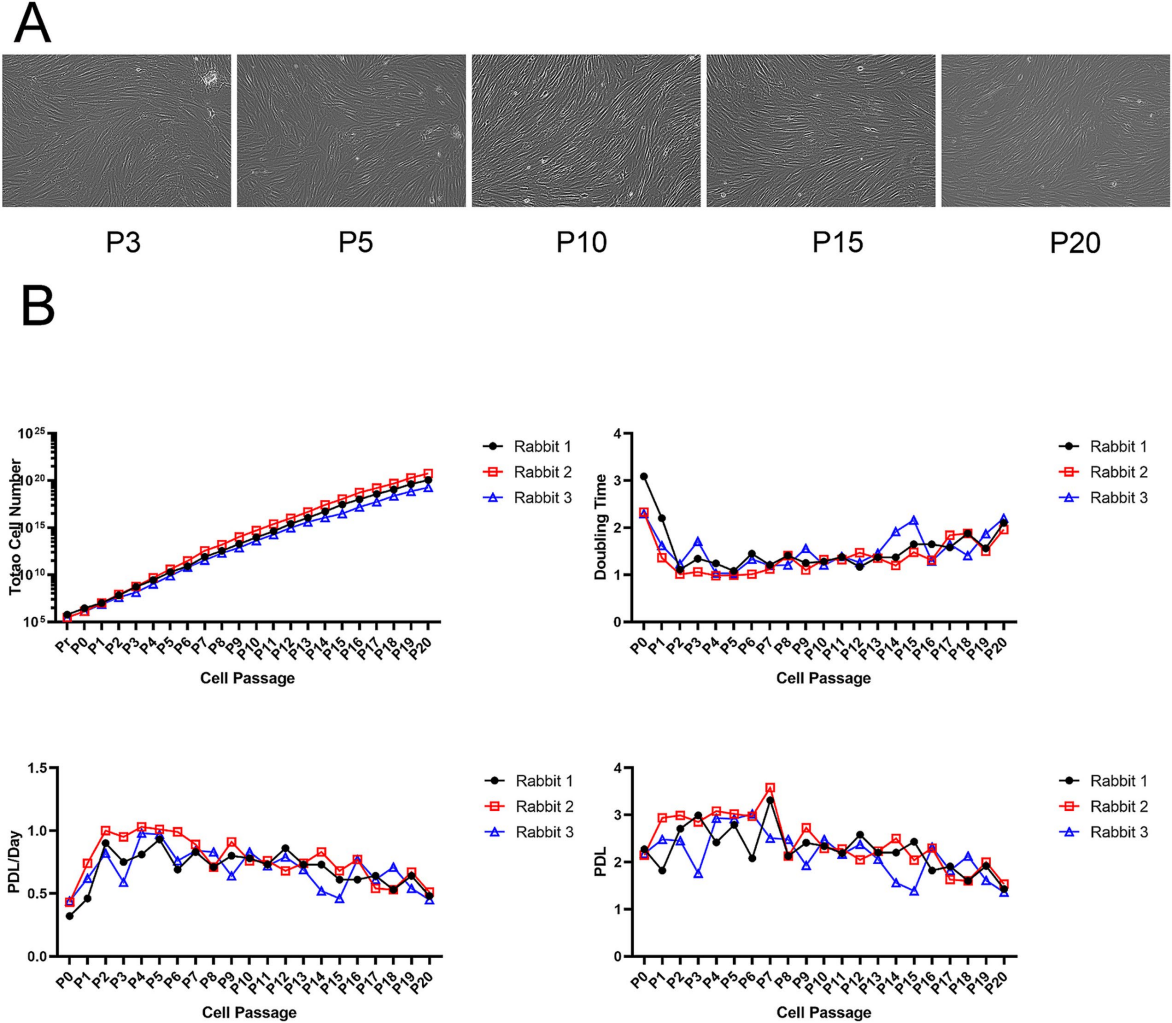
(A) Microscopy images of fibroblasts at different passages. (B) Growth kinetics of allogeneic fibroblasts.

We used allogeneic fibroblasts from normal competent donor rabbits for *in vitro* culture and expansion. After isolation, the allogeneic fibroblasts differentiated and proliferated, becoming spindle-shaped after passage. Under the microscope, rabbit allogeneic fibroblasts at P3, P5, P10, and P15 showed better appearance and morphology without contamination (Figure 1A). Three rabbits were randomly selected for autologous fibroblast preparation, showing that our method can stably extract and expand fibroblasts. Rabbit allogeneic fibroblasts showed consistent growth curves, faster growth rates, and strong cell proliferation, allowing them to be used in our subsequent cell therapy experiments (Figure 1B). However, their proliferative potential diminished significantly after 21 passages.

### 3.2 Identification of allogeneic fibroblasts

**Figure 2:**
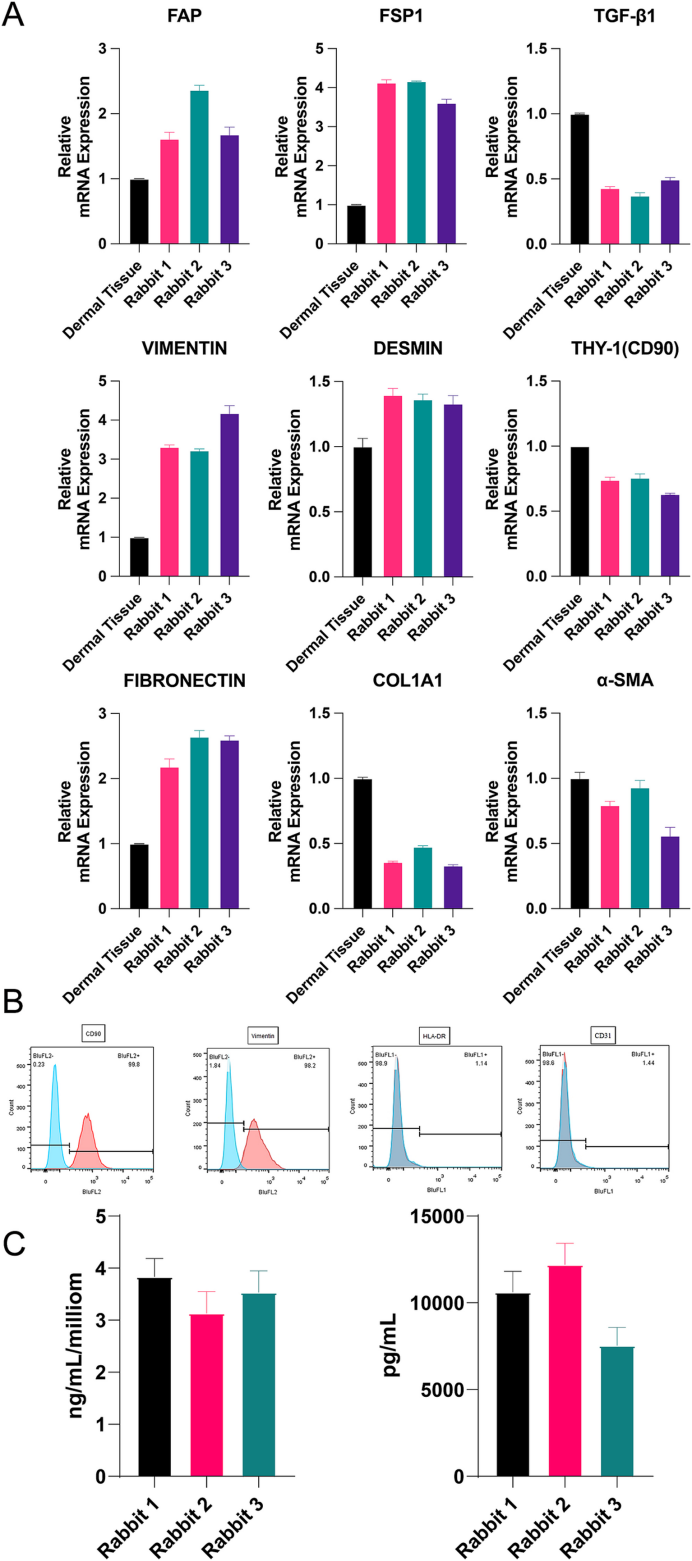
**(A)** The mRNA expression of *α*-SMA, COL1A1, FAP, THY, TGF-*β*1, S100A4, fibronectin, vimentin, and Desm. **(B)** Flow cytometry of CD9, vimentin, CD31 and HLA-DR. **(C)** Concentration of TGF-*β*1 and COL-1 by ELISA. *p < 0.05, **p < 0.01, ***p < 0.001, and ****p < 0.0001.

Previous experiments have shown that fibroblasts from the 5th passage have the best growth potential. Therefore, we extracted allogeneic fibroblasts from three rabbits to verify their cellular type, differentiation, and paracrine capacity. To characterize the phenotype of allogeneic fibroblasts, we used RT–PCR to determine the expression of relevant mRNAs. The results showed that the expression of fibroblast-related genes was stable, and the cells could proliferate *in vitro* (Figure 2A), although some genes were expressed at lower levels in the dermal tissue. In addition, flow cytometry results showed that the related antigens CD90 and Vimentin, which are labeled on the surface of fibroblasts, were highly expressed in allogeneic fibroblasts, whereas CD31 and HLA-DR were expressed at low levels, which proved that they are fibroblasts (Figure 2B). The ELISA results showed that the expanded cells stably expressed TGF-β1 and COL-1, which are indicators of good conditions (Figure 2C).

### 3.3 Allogeneic fibroblasts maintain disc height in rabbits

**Figure 3:**
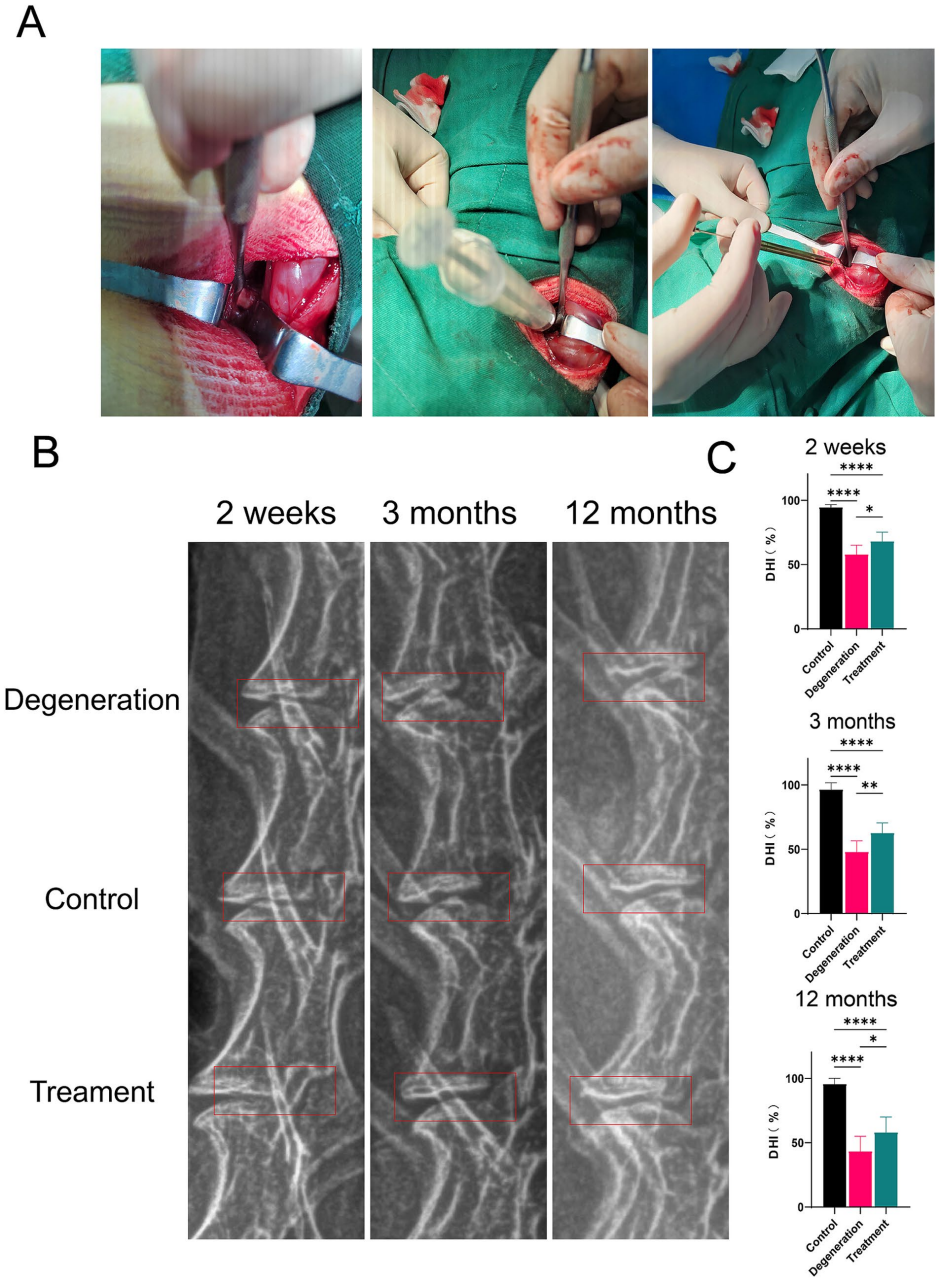
(A) Surgery photos. (B) Radiographic images. (C) The percentage of the disc height index (DHI). Error bars represent the S.D., and significant differences were assessed with Student’s *t* test; *p < 0.05, **p < 0.01, ***p < 0.001, and ****p < 0.0001.

We first exposed the intervertebral discs of rabbits and constructed metaplastic models by puncturing and injecting allogeneic fibroblasts through a microinjector (Figure 3A). Imaging and histologic observations were performed on the rabbits 2 weeks, 3 months, and 12 months after the surgery. The height of the intervertebral disc (DHI) was measured via X-ray. The percentage was calculated as (target/means of all measurements x 100). As illustrated in the red box of figure, the disc height is maintained obviously (Figure 3B). As shown in the figure, the percentage of DHI in the degeneration group (0.58 ± 0.071) was significantly lower than that in the control group (0.947 ± 0.019) at 2 weeks after surgery (*p* < 0.0001). In addition, the percentage of DHI in the treatment group (0.682 ± 0.069) was significantly greater than that in the IVDD group (*p* < 0.5). At 3 months after surgery, the percentage of DHI in the degeneration group (0.48 ± 0.085) was significantly lower than that in the control group (0.965 ± 0.051; *p* < 0.0001). In addition, the percentage of DHI in the treatment group (0.627 ± 0.078) was significantly greater than that in the IVDD group (*p* < 0.01). At 12 months postoperatively, the percentage of DHI in the degeneration group (0.433 ± 0.116) was significantly lower than the control group (0.957 ± 0.044; *p* < 0.0001) and treatment group (0.581 ± 0.119; *p* < 0.5; Figure 3C).

### 3.4 Allogeneic fibroblasts alleviate intervertebral disc degeneration

**Figure 4:**
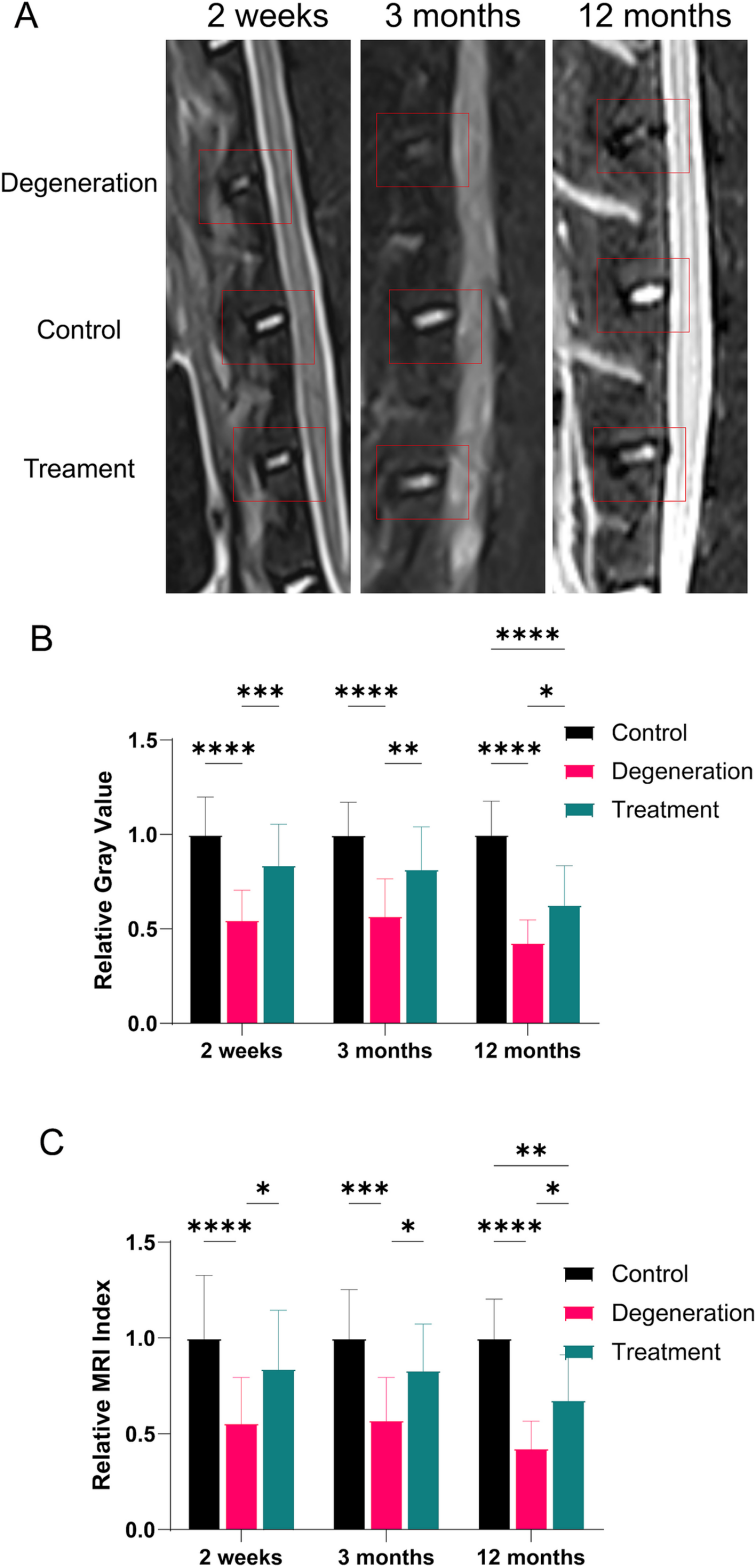
(A) MR images at 2, 3, and 12 months after surgery and treatment were acquired and measured; (B) relative gray value analysis at 2 weeks, 3 and 12 months after surgery and treatment; (C) relative MRI index analysis at 2 weeks, 3 and 12 months after surgery and treatment.

MRI can also assess the degree of disc degeneration by calculating the grayscale value and MRI index (gray value multiplied by area). MRI at 2 weeks, 3 and 12 months revealed a significant reduction in the gray value and area within the degeneration group compared to those of the control group and the treatment group (Figure 4A). After 2 weeks of recovery from surgery, the MRI results showed that the relative gray value and relative MRI index were significantly lower in the degeneration group than in the control group (*p* < 0.0001; Figures 4B,C). The relative gray value and MRI index of the treatment group were greater than those of the degeneration group (*p* < 0.5). In addition, 3 months after surgery, as shown in the figure, the gray value and MRI index of the degeneration group were lower than those of the treatment group (*p* < 0.5) and the control group (*p* < 0.001). Similarly, 12 months after surgery, as shown in the figure, the relative gray value and MRI index of the degeneration group were lower than those of the treatment group (*p* < 0.5) and the control group (*p* < 0.0001).

### 3.5 Histological analysis

**Figure 5:**
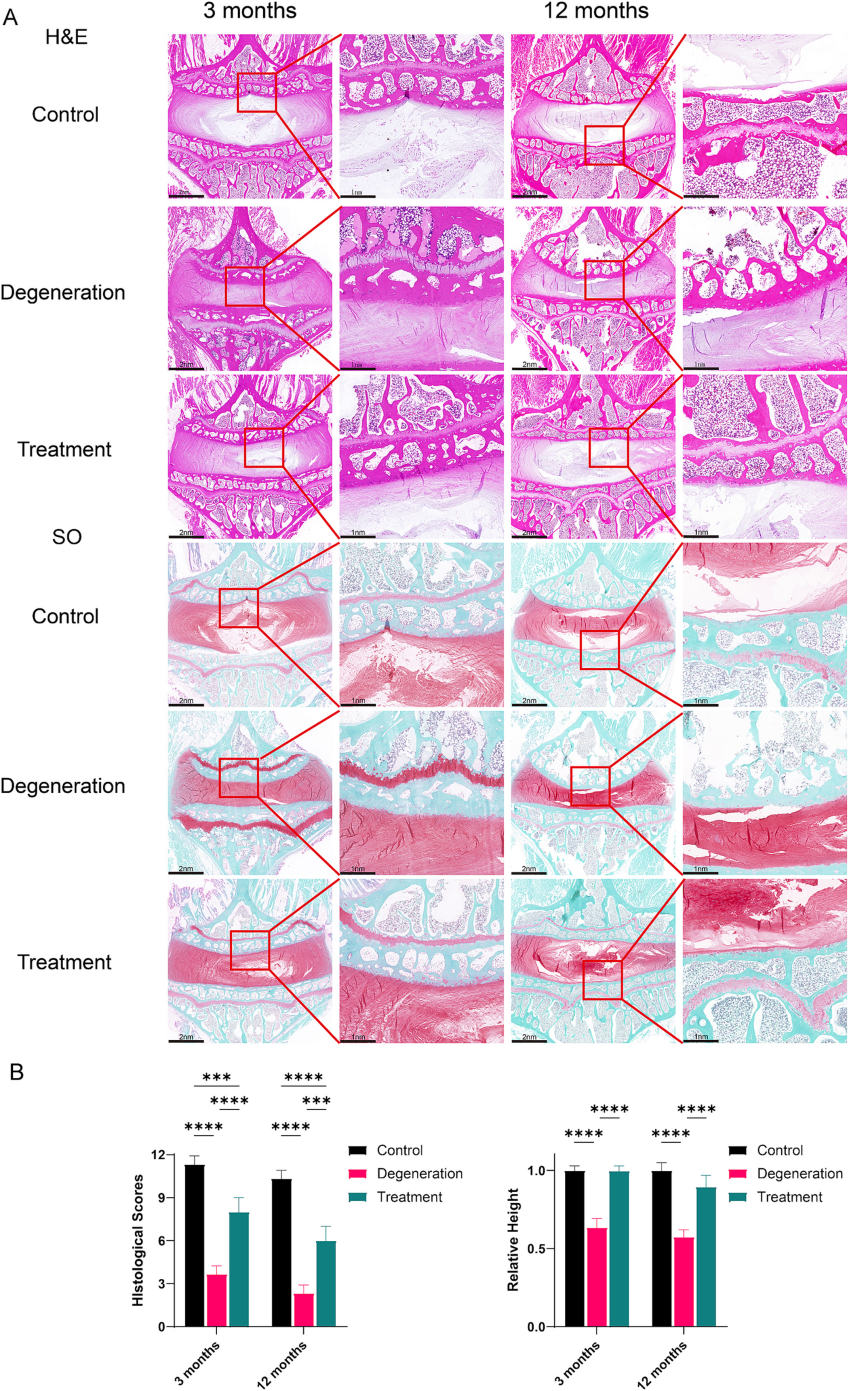
(A) H&E staining of tissue sections showing general cellular distribution and SO staining showing cartilage and proteoglycan distributions and degeneration at 3 months and 12 months. (B) Histological scoring based on SO and H&E sections and the percentage of relative height based on SO and H&E sections. Error bars represent the S.D., and significant differences were assessed with Student’s *t* test; *p < 0.05, **p < 0.01, ***p < 0.001, and ****p < 0.0001.

The results of HE and SO histochemical staining of intervertebral discs in each group showed that the intervertebral discs (IVDs) in the control group had well-structured inner gel-like NPs and outer concentric ring-like AF tissues. However, in the degeneration group, a large amount of nucleus pulposus tissue was lost, and the structure of the IVD was destroyed (Figure 5A). The experimental IVDs did not show discernible NP-AF boundaries, and structures within the IVD space appeared disordered. In the treatment group, NP was observed along with a reduction in fibrosis. The magnified image also shows that the endplate tissue, which was destroyed by the puncture, was salvaged and presented a more complete structure with more cells. The histological score of the degeneration group was significantly lower than that of the control group (*p* < 0.0001). Interestingly, the loss of nucleus pulposus in the treatment group was significantly lower than that in the degeneration group, and the histological score was greater than that in the degeneration group (*p* < 0.001; Figure 5B). In addition, the relative disc height was significantly lower in the degeneration group than in the control group (*p* < 0.0001) and the treatment group (*p* < 0.0001; Figure 5C).

### 3.6 Allogeneic fibroblasts inhibit collagen transformation and extracellular matrix breakdown in intervertebral discs

**Figure 6:**
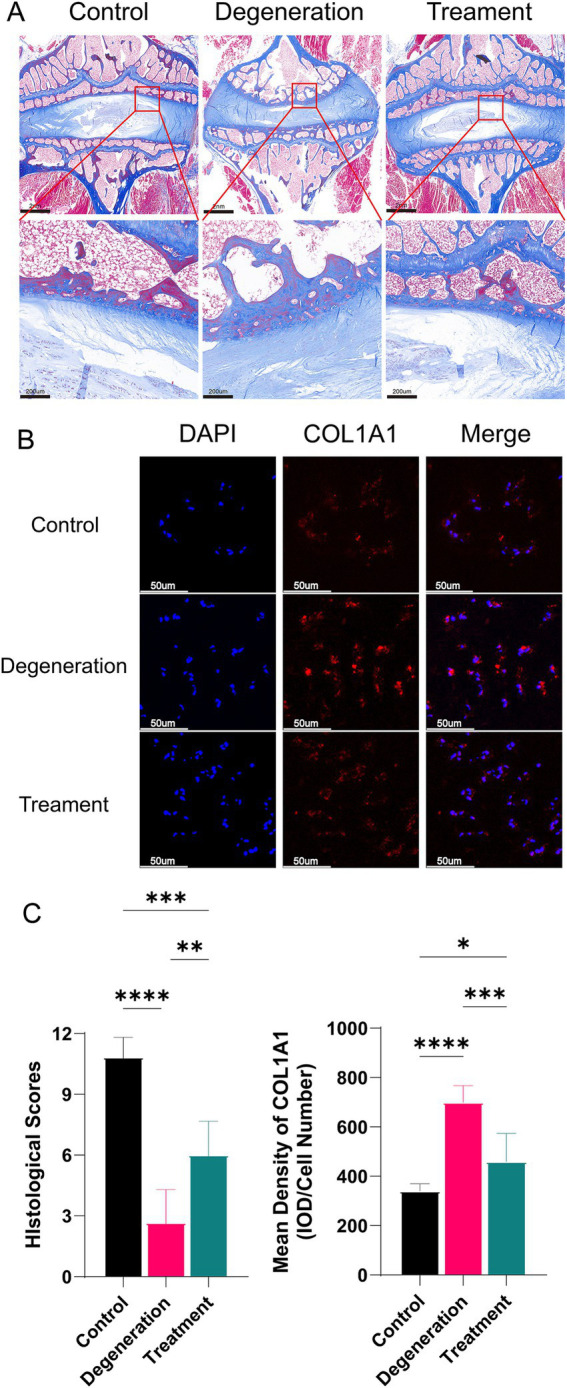
(A) Masson staining of tissue sections showing the collagen distribution at 12 months. (B) Immunofluorescence for COL1A1 in the NP. (C) Histological scoring based on Masson sections and the mean density of MMP2 (IOD/cell number). Error bars represent the S.D., and significant differences were assessed with Student’s *t* test; *p < 0.05, **p < 0.01, ***p < 0.001, and ****p < 0.0001.

The nucleus pulposus consists mainly of type II collagen, and as degeneration progresses, the collagen fiber composition within the nucleus pulposus changes, resulting in an increase in type I collagen and a decrease in type II collagen. At 3 and 12 months after surgery, Masson staining revealed a lower concentration of type I collagen in the myeloid nucleus of the treated group than in that of the degenerated group, indicating that the treated discs retained more hydrophilicity and water retention (Figure 6A). Additionally, the degeneration group exhibited a decrease in type II collagen and an increase in type I collagen notches in the endplates, whereas the treatment group showed more type II collagen and intact endplates. Immunofluorescence for COL1A1 suggested that there were fewer type I antigens in the treatment group than in the regression group (Figure 6B). After analysis, the histological score of the degeneration group was significantly lower than that of the control group, and the mean density of COL1A1 in the degeneration group was significantly greater than that in the control group (Figure 6C).

### 3.7 Allogeneic fibroblasts reduce osteoclastogenesis in intervertebral disc degeneration

**Figure 7:**
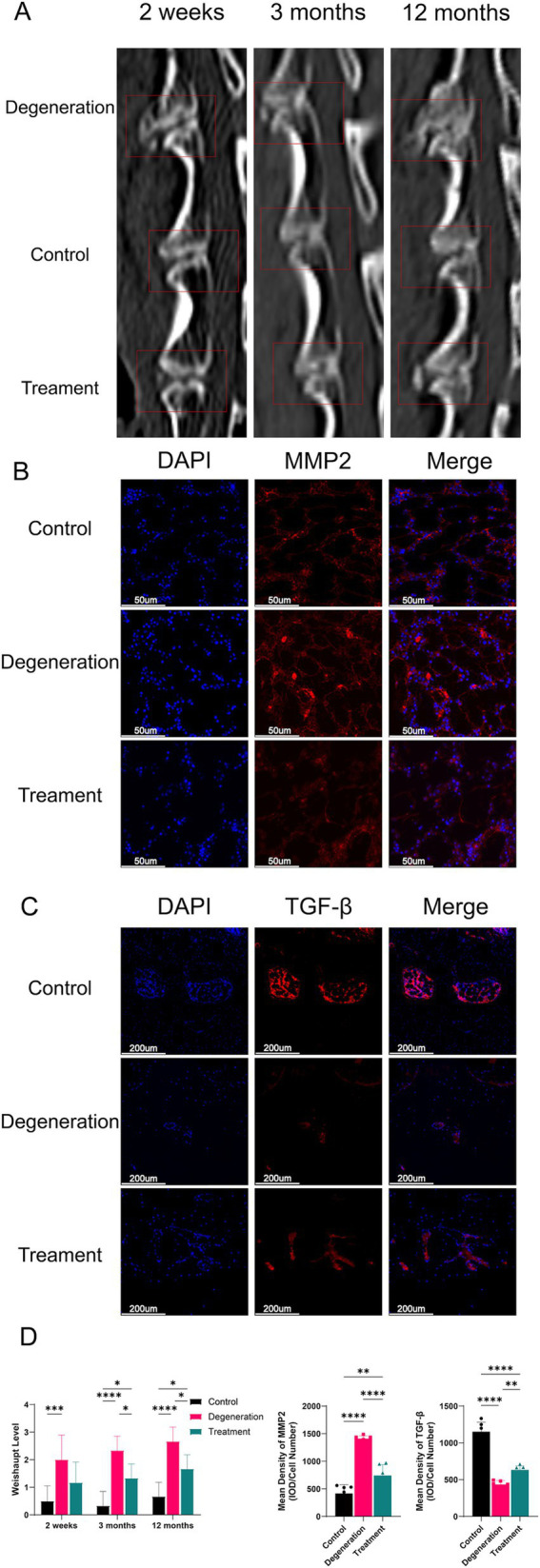
(A) CT images at 2 weeks, 3 months, and 12 months after surgery and treatment were acquired and measured; (B) Immunofluorescence for MMP2 in the endplate; (C) Immunofluorescence for TGF-β in the endplate; (D) Weishaupt level of the IVD at 2 weeks, 3 months and 12 months after surgery. Mean density of MMP2 and TGF-β (IOD/cell number). Error bars represent the S.D., and significant differences were assessed with Student’s *t* test; *p < 0.05, **p < 0.01, ***p < 0.001, and ****p < 0.0001.

During the postoperative period, we used CT to visualize the growth of the disc’s osteophytes (Figure 7A). The CT data showed that at 2 weeks postsurgery, there were significantly more osteophytes in the degeneration group than in the control group (*p* < 0.001), whereas there was no significant difference in the treatment group. At 3 months, there was significantly more osteoid production in the degeneration group than in the control group (*p* < 0.0001), while there was less osteoid production in the treatment group than in the control group (*p* < 0.5). In addition, at 12 months, the degeneration group had significantly more osteoid formation than the control group (*p* < 0.0001) and the treatment group (*p* < 0.5; Figure 7D). MMP2 is an enzyme closely associated with extracellular matrix degradation. Immunofluorescence for MMP2 suggested that there was more MMP2 in the degeneration group than in the treatment group, possibly leading to more extracellular matrix degradation and glycogenolysis (Figure 7B). TGF-*β* can promote the synthesis of a cartilage-specific matrix by inducing the differentiation of mesenchymal cells into chondrocytes. More TGF-β in the endplate of the treatment group than in the degeneration group protected the cartilage matrix from hydrolysis and destruction by various proteases (Figure 7C).

## 4 Discussion

Intervertebral disc degeneration is a multifactorial disease commonly attributed to aging, inflammation, and other environmental stresses (17, 18). New biological therapies, such as biomaterial-based tissue engineering, cellular therapy, growth factor injections, and gene therapy, show great promise in the treatment of intervertebral disc degeneration (IVDD), despite the limitations of surgical treatment (19, 20). Recent studies suggest that fibroblasts may play a crucial role in preserving disc height and preventing disc degeneration (9). However, the specific effect of fibroblasts on this process remains unclear.

There are only two cell types in the human body capable of regenerating tissue and organs: stem cells and fibroblasts (21). Fibroblasts are the main cell type of connective tissue and possess a spindle-shaped morphology. They produce and maintain the extracellular matrix responsible for the structural integrity of tissues and organs, which plays key roles in fibrosis, cancer, autoimmunity, and wound healing (22). Detailed analysis of barrier tissues, such as skin, gut, and lung, has shown that some fibroblasts directly sense pathogens and other danger signals to elicit host defense functions (23, 24). These functions include antimicrobial activity, leukocyte recruitment, and the production of cytokines and lipid mediators that are relevant to inflammation and immunosuppression. Fibroblasts are mesenchymal cells that exhibit remarkable plasticity in adapting their properties to the microenvironment’s needs (25, 26). Fibroblasts can also act synergistically with other cells, including stem cells and macrophages (27, 28).

However, many studies have proven fibroblasts to be more effective and more potent than stem cells in regeneration and immune modulation (29, 30). Although bone marrow is the most commonly used MSC source, it provides relatively little starting material for cellular expansion and requires invasive extraction methods. On the other hand, fibroblasts can be easily harvested in large numbers from various biological wastes (31). Fibroblasts, the most common cell type in the human body, are easier to source and culture (32). Compared to stem cell therapy, fibroblast-based therapies are more accessible to patients, more effective, and less expensive. There are also many fibroblast treatments currently approved by the FDA for clinical use, such as Dermagraft (33).

Therefore, this study aimed to investigate whether fibroblasts could effectively slow the degeneration of intervertebral discs. These findings suggest that fibroblasts could have potential therapeutic applications in treating IVDD. Compared with those in the degeneration group, autologous fibroblasts may delay IVDD by maintaining disc height and inhibiting osteophyte formation, as indicated by the significant improvements in the nucleus pulposus, annulus fibrosus, and osteophytes observed in the treatment group. The formation of osteophytes is closely linked to pain in patients with disc degeneration, which significantly impacts their quality of life (34, 35).

However, this study has several limitations. First, the long-term therapeutic effects and potential side effects of autologous fibroblasts in alleviating disc degeneration are still unknown. Second, the data in this study were based on a small number of animals, which calls for further research with larger samples. Third, although our *in vivo* results are promising, it is essential to validate these findings *in vitro*.

## 5 Conclusion

Our data indicate that stable and reliably extracted allogeneic fibroblasts can effectively ameliorate intervertebral disc degeneration by reducing osteophytes, which casts a new light on cell therapy for intervertebral disc degeneration. Results provide a basis for advancing the further translation of fibroblasts in intervertebral disc therapy.

## Data Availability

The original contributions presented in the study are included in the article/supplementary material, further inquiries can be directed to the corresponding author.
